# Anti-Müllerian hormone beyond an ovarian reserve marker: the relationship with the physiology and pathology in the life-long follicle development

**DOI:** 10.3389/fendo.2023.1273966

**Published:** 2023-11-03

**Authors:** Akira Iwase, Yuko Hasegawa, Yumiko Tsukui, Mio Kobayashi, Hikaru Hiraishi, Tomoko Nakazato, Yoshikazu Kitahara

**Affiliations:** Department of Obstetrics and Gynecology, Gunma University Graduate School of Medicine, Maebashi, Japan

**Keywords:** anti-Müllerian hormone, follicle cohort, oocyte, ovarian reserve, puberty

## Abstract

Anti-Müllerian hormone (AMH), an indirect indicator of the number of remaining follicles, is clinically used as a test for ovarian reserve. Typically, a decline suggests a decrease in the number of remaining follicles in relation to ovarian toxicity caused by interventions, which may implicate fertility. In contrast, serum AMH levels are elevated in patients with polycystic ovary syndrome. AMH is produced primarily in the granulosa cells of the preantral and small antral follicles. Thus it varies in association with folliculogenesis and the establishment and shrinking of the follicle cohort. Ovarian activity during the female half-life, from the embryonic period to menopause, is based on folliculogenesis and maintenance of the follicle cohort, which is influenced by developmental processes, life events, and interventions. AMH trends over a woman’s lifetime are associated with *in vivo* follicular cohort transitions that cannot be observed directly.

## Introduction

Ovarian function can be summarized as 1) the maturation of the oocyte to a state where fertilization is possible and 2) the production of female hormones. These are mainly carried out by the oocyte itself, granulosa cells, and theca cells, all of which are components of the follicle. Therefore, ovarian function is the functional sum of the follicle.

It is believed that the main factor in the decline in fertility with age is the qualitative and quantitative decline in oocytes. In other words, the concept of ovarian reserve emerged because it was thought that an index that reflects the quantity and quality of follicles in the ovary could be used to assess its impact on female reproductive function (1). However, because the qualitative evaluation of individual oocytes leading to pregnancy and live birth remains difficult for oocytes *in vivo*, the ovarian reserve has been established as a quantitative indicator of the oocytes remaining in the ovary (2).

Research on the usefulness of anti-Müllerian hormone (AMH) as an ovarian reserve test began in 2002, and it was reported that serum AMH levels decline with age in women with normal ovulatory cycles, correlating with antral follicle count and age (3). Subsequently, several clinical applications have been developed (4).

AMH is produced by follicles at a specific developmental stage and not secreted by primordial follicles; therefore, it does not directly reflect the number of follicles remaining in the ovary. The oocytes present in the ovary form a pyramid called the follicular cohort that decreases in number as the follicles mature. Therefore, AMH is an ovarian reserve marker that indirectly reflects the number of oocytes remaining in the ovary. Thus, AMH levels change, influenced by follicular cohort formation and development. This study examined the association between AMH changes and clinical utility in terms of follicular cohorts during a woman’s lifetime.

## AMH action and follicle growth

AMH, also called Müllerian inhibiting substance, is a hormone or cytokine derived from its first identified function in fetal sex differentiation. AMH produced in the fetal testis causes retraction of the Müllerian ducts during sexual differentiation into males during embryonic life. In humans (5, 6), it has been reported to be expressed in early-stage developing follicles in adult female ovaries (7), whereas in fetuses, it has been detected only in the granulosa cells of follicles after approximately 25 weeks of fetal life (8). The uterus continues to grow during the late embryonic period, but the uterine primordium, which arises from the Müllerian duct, is already complete by this time.

The effects of AMH on ovaries have been demonstrated in experiments using AMH-knockout mice by Durlinger et al. In AMH knockout mice, there are no significant changes in female fertility; however, the recruitment of primordial follicles is enhanced, resulting in early follicle depletion (9). In other words, AMH suppressed primordial follicle recruitment. Furthermore, AMH suppresses FSH-dependent follicle development (10). It also inhibits estrogen production by granulosa cells during FSH-dependent follicle development by decreasing FSH sensitivity and suppressing aromatase induction (11). The effects of AMH on the recruitment of AMH-producing stage follicles and suppression of development to the upper stages suggest that AMH plays an important role in the maintenance of the follicle cohort.

## AMH production in oogenesis and folliculogenesis

In humans, oogenesis and folliculogenesis consist of three phases: 1) initiation of meiosis and formation of primordial follicles, 2) contribution of folliculogenesis to cohort formation before puberty, and 3) establishment of a follicle cohort related to cyclic ovulation (2). In the first step, primordial germ cells proliferate into oogonia and develop into primary oocytes, followed by oocyte nest formation. The breakdown of nests that occurs during the second trimester in humans results in the formation of primordial follicles with pregranulosa cells (12). AMH-positive primary follicles have been identified in the ovaries of 25-week fetuses, and serum AMH levels were measurable in preterm-born infants at the equivalent of 26 weeks (8). With the appearance of primordial follicles after oocyte nest breakdown, follicle cohort formation begins, albeit immaturely. AMH is produced from follicles in the developing stage, and serum AMH can be measured from the neonatal period (13).

The significance of serum AMH measurement as an ovarian reserve test during the neonatal period is unknown because a follicular cohort associated with the ovulatory cycle has not yet been established. However, several studies have focused on neonatal AMH concentrations and their relation to polycystic ovary syndrome (PCOS). A meta-analysis of six observational studies demonstrated that serum AMH levels were significantly higher in neonates of mothers with PCOS compared to those of healthy controls (14). Although the implications of elevated serum AMH levels in neonates are unclear, the results may support the hypothesis that the intrauterine environment affecting ovarian function is involved in the pathogenesis of PCOS.

**Figure 1:**
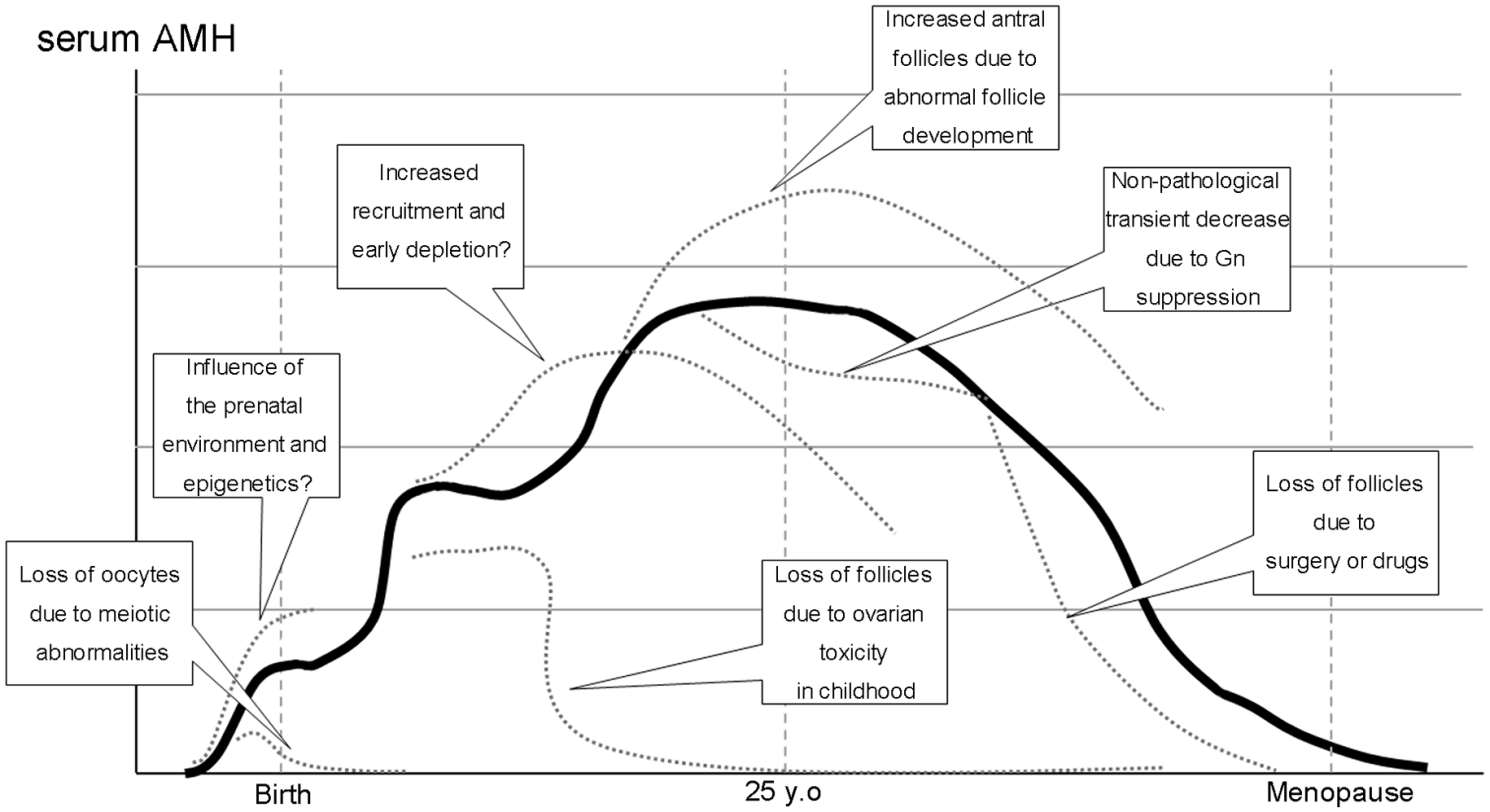
Normal (solid line) and pathological conditions (dotted lines) in folliculogenesis and serum AMH levels during a woman’s half-life.

In animal models, prenatal exposure to several hormones has been associated with postnatal PCOS phenotypes (15). Excessive androgen exposure during the prenatal period has been implicated in alterations in AMH expression and may cause abnormal folliculogenesis (16). Recently, exposure to excess AMH during pregnancy has been shown to affect the maternal brain-ovary cascade, resulting in maternal androgenization (17). However, it is not clear how environmental changes during the fetal period affect oocyte nest breakdown and, consequently, how they affect follicular cohorts. Exposure to androgens during the embryonic period has been reported to affect systemic organs via epigenetics (18). DNA methylation is also involved in regulating follicle development (19). Therefore, the embryonic environment may affect folliculogenesis and ovarian reserve through follicle cohort regulation after birth (Influence of the prenatal environment and epigenetics in **Figure 1**).

## Meiotic error and ovarian reserve

Several pathological conditions may cause the loss of follicles and lead to primary or secondary amenorrhea, including premature ovarian insufficiency (POI). Follicle formation is also a progressive process of meiosis, during which the loss of oocytes due to chromosomal abnormalities can occur. Apoptosis caused by the failure of chromosomal pairing in meiosis I is believed to be the underlying mechanism (Loss of oocytes due to meiotic abnormalities in **Figure 1**.).

Turner syndrome (TS) is the most prevalent of these conditions. Women with TS exhibit prenatal or postnatal loss of follicles depending on their karyotypes. While the loss of an X chromosome (45, X) results in streak gonads that exhibit complete loss of follicles, women with mosaicism, including normal chromosomal sets such as 45, X/46, XX, may exhibit spontaneous puberty (20, 21). In contrast, independent of karyotype, AMH appeared to be a reliable marker for predicting oocyte cryopreservation success (22). Moreover, higher AMH levels in TS have been reported to be associated with spontaneous puberty and a lower FSH (23). Lunding et al. reported that serum AMH levels below a certain threshold may be useful for predicting the absence of puberty and POI (24). Although longitudinal data regarding serum AMH levels in TS are limited, oocyte cryopreservation should be considered for adolescent TS impending premature ovarian failure regardless of karyotypes (22, 25).

## Effects of mini-puberty on ovarian reserve

The next period of change in AMH production related to follicle development occurs approximately 2 years after birth. This period is considered to correspond to mini-puberty. However, changes in females during this period are less pronounced and appear later compared to those in males (26). Gonadotropins and sex steroids increase during mini-puberty, which corresponds to 6 months in boys and 2 to 4 years in girls. Transient increases in gonadotropins during mini-puberty stimulate follicular growth into antral follicles, followed by an increase in AMH and sex steroids (8). Although AMH seems to increase until approximately 8 years of age, serum levels of AMH are more stable than those of other reproductive hormones in the prepubertal periods (27). Unlike boys, the significance of mini-puberty in girls is not clear, and it is not conclusive how changes in the follicular cohort during this period affect the future ovarian reserve.

## Establishment of follicle cohort accompanying cyclic ovulation

The subsequent period that influences follicular development is puberty, which includes the period of prepubertal follicle formation and the subsequent ovulatory cycles. Although gonadotropin secretion is low during the prepubertal stage, follicles have established their development into the gonadotropin-sensitive stage. AMH is measurable but low at this stage, as shown in a cross-sectional study (28). The process of establishing a follicular cohort, followed by cyclic menstruation, begins with an increase in gonadotropins during puberty (29). The decline in AMH, correlating with the transition during the prepubertal-pubertal phase, indicated that follicle development had entered the gonadotropin-dependent phase. Follicles in the AMH-producing stage develop into later stages in which AMH is not produced. However, follicle development at this stage is not accompanied by sufficient FSH stimulation, leading to ovulation. Subsequently, the serum AMH levels transiently decreased. Given that AMH suppresses recruitment from primordial follicles, this transient decrease in AMH levels may encourage increased recruitment to establish a follicular cohort for cyclic ovulation.

Central precocious puberty is induced by gonadotropin-stimulated ovarian estrogen secretion. Follicular development should also occur at this time; however, the behavior of AMH levels is not conclusive (13). In female precocious puberty, serum AMH levels have been reported to be similar to those in healthy controls, which suppress GnRH analogue therapy (30). Although AMH production declines, estradiol production increases owing to the development of AMH-producing follicles to the upper stage; however, it is not expected to show a constant increase because it does not lead to an oscillation in cyclic follicle development. Jeffery et al. confirmed the AMH model in girls, showing a modest peak around 9 years of age followed by a weak decrease at 10–12 years of age, just before menarche. They also showed the inverse relationship between AMH and FSH before puberty that disappears at the onset of puberty (31). Although the drive for this change has not been fully established, it may be explained by the different modes of gonadotropin control over the two processes of follicle development, that is, recruitment from the primordial follicle to the growing follicle pool and development from AMH-producing follicles to AMH-nonproducing follicles. Specifically, serum estradiol levels gradually increase during the prepubertal years, possibly corresponding to a transient decrease in AMH (32).

## Excess recruitment and impairment ovarian reserve

There are two periods from embryonic development to menopause, during which AMH production increases in aggregates. The first increase, which initiates AMH production, occurs at the time of primordial follicle emergence, following oocyte nest breakdown. The second increase occurred at the time of follicle cohort establishment during ovulatory cycle formation during puberty, as described earlier. The details of how this period influences the control of follicle development, and thus, ovarian reserve, are not yet known.

Hashimoto disease has been reported to be associated with POI. Endocrine abnormalities and impairment of follicular growth due to autoimmunity have been postulated; however, the details of this mechanism are unknown (33). A meta-analysis of age stratification using AMH level as an indicator reported interesting results. Hasegawa et al. demonstrated that AMH levels were substantially higher in adolescents and young adults with thyroid antibodies, while AMH levels tended to decline in all age groups (34). Extensive recruitment of primordial follicles may occur under autoimmune conditions and follow the atresia of damaged growing follicles (Increased recruitment and early follicle depletion in **Figure 1**).

At this stage, it is difficult to conclude whether over-recruitment leads to the subsequent loss of ovarian reserve in humans. In experimental animals, AMH knockout has been shown to result in increased primordial follicle recruitment, leading to early depletion (9). The burnout theory with cyclophosphamide, discussed below (35), is also a similar concept. However, cyclophosphamide can impair follicles at all developmental stages. Although follicle damage caused by autoimmunity has long been a matter of debate (36, 37), no studies have clearly demonstrated an association with follicle cohort-regulated AMH, which is an issue for future research.

## Clinical implication of AMH in adolescent and young adult

Considering the actions of AMH, inhibition of primordial follicle recruitment, and FSH-dependent development, AMH production from the established follicle cohort may maintain dynamic stability in this cohort. Late puberty through the early 20s is the period of the least longitudinal change in AMH levels (23, 28). Several ovulation disorders, including PCOS, occurred relatively frequently during this period. Ovulatory failure in PCOS is caused by a hyperandrogenic environment and relatively insufficient FSH action and is characterized by arrested development at the small antral follicle stage (Increased antral follicles due to abnormal follicle development in **Figure 1**). Hence, serum AMH levels in women with PCOS are higher than those in women with normal ovulation. Although meta-analyses have confirmed that women with PCOS of all ages have higher AMH values than age-matched control women (38, 39), it is difficult to establish a universal AMH cut-off because AMH levels decline in an age-dependent manner (40).

Relatively fewer age-dependent changes in serum AMH occur between late adolescence and the early twenties when the ovulatory cycle is established and the AMH-producing follicles are in equilibrium. Therefore, it would be helpful to understand pathophysiology based on absolute AMH values. Tsukui et al. reported in a meta-analysis that AMH is useful as an adjunct diagnosis for PCOS in this age group and that its cutoff can be relatively narrowed (41).

In this age group, differentiating PCOS from functional hypothalamic amenorrhea (FHA) has become a clinical issue considering that polycystic ovarian morphology—a PCOS triad—is often found in young FHA women (42). Although differential diagnosis is not difficult based on clinical findings such as body weight and hyperandrogenism, AMH levels may also be useful. FHA is caused by the impairment of the hypothalamus-pituitary system, resulting in decreased gonadotropin secretion and anovulation due to the absence of gonadotropin-dependent follicular development. In addition, persistently low gonadotropin levels cause a decrease in the number of AMH-producing follicles, in which growth is regulated in a gonadotropin-sensitive manner.

## AMH decrease after establishment of follicle cohort: physiological conditions

After 25 years of age, AMH levels declined progressively. The follicular cohort is maintained, but as the follicles at each stage gradually become atretic, the cohort shrinks; consequently, the number of follicles in the AMH-producing stage also decreases. Several short-term life events may result in a transient decline in serum AMH levels. Women with low AMH levels show little variation within the menstrual cycle, whereas women with high AMH levels, such as younger women, tend to have slightly higher levels in the follicular phase, slightly lower levels in the ovulatory phase, and recover in the luteal phase (43, 44). This is based on a transient decrease in the number of follicles in AMH-producing stages as the follicular cohort moves in a developmental direction. This trend was more pronounced after ovarian stimulation with gonadotropins.

As follicle growth is inhibited during pregnancy with increased sex steroids, AMH levels decrease over the course of pregnancy, with significant decreases in AMH levels in the second and third trimesters compared to the first trimester, with an average decrease of 50% at the end of pregnancy reported (45). It has also been reported that values begin to increase approximately 4 days after delivery (46).

Oral contraceptive (OC) use decreases AMH levels (47). In a cohort study of 863 women (228 OC takers and 504 non-OC takers), serum AMH levels were 29.8% lower in OC takers than in non-OC-takers (48). This decrease in serum AMH was based on the suppression of the follicular cohort by gonadotropin suppression caused by the exogenous administration of sex steroid derivatives, similar to the FHA and pregnancy described above; however, the total number of follicles, including primordial follicles, was not reduced (Non-pathological transient decrease due to Gn suppression in **Figure 1**).

## AMH decrease after establishment of follicle cohort: ovarian toxicity

AMH levels can decrease due to anticancer drugs at any age (49). However, AMH production may differ depending on whether anticancer drugs are used before or after the establishment of the follicle cohort. In cases where the follicle cohort has already been established, it is proposed that a cycle begins in which developing follicles, which are more sensitive to anticancer drugs, are damaged, resulting in decreased AMH production. The decrease in AMH thereby enhances the recruitment of primordial follicles, which are similarly damaged by anticancer drugs because of the increase in gonadotropins caused by the lack of negative feedback from the decrease in sex steroids. This mechanism is called the burnout theory (35). In childhood, on the other hand, a follicular cohort exists but has not yet been established, so it is assumed that the burnout cycle induced by AMH reduction due to developmental follicle damage is less likely to be involved.

Several studies in pediatric populations (i.e., under 18 years of age at cancer diagnosis) did not detect a significant difference in post-treatment AMH versus controls despite long-term follow-up periods of 10–30 years in heterogeneous populations of childhood cancer survivors (50–52). This may be due to the difference in the mechanisms mentioned above; however, even in childhood, severe damage to the primordial follicle can result in follicle depletion and cause permanent menopause or diminished ovarian reserve (DOR) after the recovery of the menstrual cycle (53) (Loss of follicles due to ovarian toxicity in childhood in **Figure 1**).

In most cases, AMH levels transiently decline after anticancer therapy. The persistence of undetectable serum AMH levels after treatment is useful as a predictor of continuous AMH deficiency re-elevation and menstrual recovery (54). On the other hand, the evidence is not sufficient to conclude whether prediction of permanent POI at the end of treatment may be possible by pretreatment AMH levels (55), although this has been reported to be possible in early-stage breast cancer (56). Further studies are needed to determine which patients and treatments may or may not be feasible.

For endometriosis, it is assumed that a decrease in ovarian reserve due to follicular damage by local inflammation is similar to but weaker than that of anticancer drugs. Kitajima et al. reported that fibrosis of the ovarian cortex due to inflammation during endometriosis may damage follicles, leading to decreased AMH production and increased recruitment of follicles (57). However, longitudinal data on AMH levels under these conditions have not been elucidated in detail.

The effect of surgery is thought to be even more direct owing to the volume reduction of the ovarian cortex (58). Comparing endometriotic cyst and benign ovarian cyst surgery, Kitajima et al. reported that the decrease in AMH was significant in the former group, with more unintended removal of the ovarian cortex in the explanted specimen (59). In contrast, Iwase et al. found that the degree of adhesion and surgical details were involved in the difference in AMH recovery after endometriotic cyst surgery. They speculated that blood flow at the time of re-establishment of the follicular cohort was affected (60). Although there are no reports quantitatively evaluating blood flow to the adnexal area and relating it to AMH variations, the possibility of a relationship between ovarian blood flow through the fallopian mesentery and the follicular cohort cannot be ruled out, based on the results of a meta-analysis evaluating the effect of tubal ablation on AMH levels (61) (Loss of follicles due to surgery and drugs in **Figure 1**).

## Increase of AMH production

Elevated AMH levels have been reported in several tumors, including granulosa cell tumors (GCTs), wherein tumorigenesis results in the growth of AMH-producing cells. GCTs are the most common subtype of ovarian sex cord-stromal tumors, accounting for 2–5% of all ovarian cancers (62). GCTs are divided into two subtypes. The juvenile type occurs primarily in children, while the adult type typically occurs in postmenopausal women.

The usefulness of AMH as a tumor marker has been reported in numerous studies. In a recent meta-analysis evaluating the performance of AMH in the diagnosis of GCT, the pooled sensitivity was 89%, with a pooled specificity of 93% (63).

## Conclusions

Much is unknown about follicle formation and follicle cohort establishment in the human ovary. Since oocytes are not reproduced after birth, the process of follicle cohort establishment from birth to puberty and sexual maturity may affect the subsequent ovarian reserve (**Figure 1**). Although follicles in the developmental stage below the small antral follicle are difficult to observe *in vivo*, follicles at this stage, excluding primordial follicles, producing AMH. Thus, AMH measurement as an ovarian reserve test provides clues to dynamic changes in the follicle cohort through an indirect estimation of the total number of remaining follicles. Longitudinal AMH measurements provide clues regarding the lifetime of the ovary and may provide clues for future interventions for ovarian longevity.

## Author contributions

AI: Conceptualization, Writing – original draft. YH: Resources, Visualization, Writing – review & editing. YT: Resources, Visualization, Writing – review & editing. MK: Resources, Visualization, Writing – review & editing. HH: Resources, Visualization, Writing – review & editing. TN: Resources, Visualization, Writing – review & editing. YK: Supervision, Writing – review & editing.
